# Supplementary material for: Navigating interdisciplinary coastal research in the UK: Challenges and solutions from an early career perspective
Source: Camb Prism Coast Futur. 2026 Jan 8;4:e2. doi: 10.1017/cft.2025.10022 (PMC12895440; doi:10.1017/cft.2025.10022)
Supplement: Apine et al. supplementary material [file S275472052510022Xsup001.docx]

Supplementary Material: Navigating Interdisciplinary Coastal Research in the UK: Challenges and Solutions from an Early Career Perspective

Online survey

**Q1** Are you an early career researcher (ECR)? For the purpose of this study we consider an early career researcher as someone who has completed their PhD qualification and has no more than 10 years of research experience.

- Yes (1)
- No (2)

**Q2** How many years post-PhD are you?

________________________________________________________________

**Q3** What is your broad research field?

- Natural sciences (e.g. biology) (1)
- Physical sciences (e.g. oceanography) (2)
- Social sciences (e.g. human geography) (3)
- Interdisciplinary research (4)

**Q4** In what discipline was your PhD?

________________________________________________________________

**Q5** How would you describe your field/profession now?

________________________________________________________________

**Q6** Which habitats/systems do you focus on?

- Coastal (1)
- Marine (2)
- Both coastal and marine (3)
- Coastal, marine and terrestrial (4)
- Terrestrial only (5)

**Q7** Would you describe yourself as an interdisciplinary researcher?

- Yes (1)
- No (2)
- Other (3) __________________________________________________

**Q8** Have you been or currently are involved in interdisciplinary research project with focus on coastal and marine habitats/communities?

- Yes (1)
- No (2)

Skip To: Q9 If Have you been or currently are involved in interdisciplinary research project with focus on coast... = No

**Q9** Would you like to be involved in interdisciplinary research projects?

- Yes (1)
- Maybe (2)
- No (3)

Display This Question:

If Would you like to be involved in interdisciplinary research projects? = Yes

**Q10** What has limited your involvement in interdisciplinary research so far?

________________________________________________________________

Display This Question:

If Would you like to be involved in interdisciplinary research projects? = No

**Q11** What are the reasons for not wanting to conduct interdisciplinary research or be part of interdisciplinary project teams?

________________________________________________________________

Display This Question:

If Would you like to be involved in interdisciplinary research projects? = Maybe

**Q12** What could help you decide?

_______________________________________________________________

**Q13** Which of the following barriers/challenges have you encountered in interdisciplinary coastal research as an ECR? (select all that apply)

- Demanding workload (1)
- Limited institutional support (2)
- Ineffective supervisory support and communication between ECR and senior scientists (3)
- Language barrier between researchers from different disciplines (4)
- Language barriers between multinational ECRs (5)
- Different research philosophies and steep learning curve (6)
- Lack of jobs and highly competitive nature (7)
- Short-term contracts (8)
- Stress because of extra pressure to network and publish (9)
- Limited guidance available for interdisciplinary students and early career researchers (10)
- Lack of available and suitable funding (11)
- Interdisciplinary researchers viewed as ‘Jack of all trades’ (12)
- Data-related barriers - limited access to data sets making ECRs to rely on senior researchers (13)
- Lack of recognition of interdisciplinary research (14)
- Difficulty to find relevant academic journals (15)
- Other (16) __________________________________________________

**Q14** Which, in your opinion, are the most prevalent barriers/challenges ECRs face in interdisciplinary research? Rank them from 1=the most prevalent to x=least prevalent.

______ Demanding workload (1)

______ Limited institutional support (2)

______ Ineffective supervisory support and communication between ECR and senior scientists (3)

______ Language barrier between researchers from different disciplines (4)

______ Language barriers between multinational ECRs (5)

______ Different research philosophies and steep learning curve (6)

______ Lack of jobs and highly competitive nature (7)

______ Short-term contracts (8)

______ Stress because of extra pressure to network and publish (9)

______ Limited guidance available for interdisciplinary students and early career researchers (10)

______ Lack o available and suitable funding (11)

______ Interdisciplinary researchers viewed as 'Jack of all trades' (12)

______ Data-related barriers - limited access to data sets making ECRs to rely on senior researchers (13)

______ Lack of recognition of interdisciplinary (14)

______ Difficulty to find relevant academic journals (15)

______ Other (please specify) (16)

**Q15** Are there any additional barriers/challenges you would like to mention?

________________________________________________________________

**Q16** Would you like to share any additional comments or examples on barriers/challenges ECRs face in interdisciplinary research?

________________________________________________________________

**Q17** What do you think are the causes/reasons behind these barriers? (select all that apply)

- Lack of experience with working on an interdisciplinary project (1)
- Lack of sufficient funding for additional training and personnel (2)
- Time consuming procedures (3)
- Lengthy process to develop interdisciplinary projects from finding collaborators and developing project plans (4)
- Lack of established networks within research centers (5)
- Conflicts of interests between the scientific community, universities, institutes, researchers, and project stakeholders (6)
- Lack of common framework in open and online data platforms that enable interdisciplinary usage of data (7)
- Other (8) __________________________________________________

**Q18** Which are the most prevalent causes for barriers/challenges ECRs face in interdisciplinary research? Rank them from 1=the most prevalent to x=least prevalent.

______ Lack of experience with working on an interdisciplinary project (1)

______ Lack of sufficient funding for additional training and personnel (2)

______ Time consuming procedures (3)

______ Lengthy process to develop interdisciplinary projects from finding collaborators and developing project plans (4)

______ Lack of established networks within research centers (5)

______ Conflicts of interests between the scientific community, universities, institutes, researchers, and project stakeholders (6)

______ Lack of common framework in open and online data platforms that enable interdisciplinary usage of data (7)

______ Other (8)

**Q19** Are there any additional causes/reasons you would like to mention?

________________________________________________________________

**Q20** Would you like to share any additional comments or examples on causes of barriers for interdisciplinary research?

________________________________________________________________

**Q21** Which solutions are the most required?

- Have contingency plans and flexibility in approach and methodology (1)
- Social opportunities with peers in other research groups to build rapport, and familiarise one another with research diversity within and between groups (2)
- Regular meetings to engage and share skills within the interdisciplinary group (3)
- Work packages/tasks should have joint leadership, with people from different disciplines to ensure better integration of tasks throughout the project (4)
- Have interdisciplinary centres/programmes that support and coordinate projects (5)
- Train supervisors (6)
- Share power horizontally (7)
- Shift from project funding to long-term funding (8)
- Focus on transparency when sharing information and knowledge (9)
- Investment in applied interdisciplinary research (10)
- Other (11) __________________________________________________

**Q22** Which solutions would you prioritise? (Order them from 1=the highest priority, x= the lowest priority)

______ Have contingency plans and flexibility in approach and methodology (1)

______ Social opportunities with peers in other research groups to build rapport, and familiarise one another with research diversity within and between groups (2)

______ Regular meetings to engage and share skills within the interdisciplinary group (3)

______ Work packages/tasks shold have joint leadership, with people from different disciplines to ensure better integration of tasks throughout the project (4)

______ Have interdisciplinary centres/programmes that support and coordinate projects (5)

______ Train supervisors (6)

______ Share power horizontally (7)

______ Shift from project funding to long-term funding (8)

______ Focus on transparency when sharing information and knowledge (9)

______ Investment in applied interdisciplinary research (10)

______ Other (11)

**Q23** Would you like to share any additional comments or examples on potential solutions?

________________________________________________________________

**Q24** What benefits in your opinion does interdisciplinary research have for coastal research?

________________________________________________________________

**Q25** Do you think interdisciplinary research is beneficial for your career progression?

- Yes - why? (1) __________________________________________________
- No - why? (2) __________________________________________________
- Other -why? (3) __________________________________________________

**Q26** Are you involved in any of the Sustainable Management of Marine Resources project as a team member?

- Yes - CoOpt (1)
- Yes - MSPACE (2)
- Yes - ROCC (3)
- Yes - ReSOW (4)
- Yes - DMV (5)
- Yes - PoL (6)
- No (7)

**Q27** Are you part of any other formal interdisciplinary network?

- Yes – which one?
- No

**Q28** How old are you?

- 18-24 (1)
- 25-34 (2)
- 35-44 (3)
- 45-54 (4)
- Above 55 (5)

**Q29** Gender

- Male (1)
- Female (2)
- Non-binary / other (3)
- Prefer not to say (4)

**Q30** What type of contract do you have?

- Fixed term (1)
- Open-ended/Permanent (2)
- Tenure-track (3)
- Other (please specify) (4) __________________________________________________

**Q31** Does your contract includes teaching?

- Yes - education focused position (around 80%) (1)
- Yes - around 20-40% for teaching (2)
- No - but I seek teaching opportunities (3)
- No - research only (4)
- Other (5) __________________________________________________

**Q32** How many institutions have you been employed by since your PhD awarded?

______________________________________________

**Q33** Have you been on maternity/paternity leave?

- Yes (1)
- No (2)

**Q34** In which country are you based?

- England (1)
- Scotland (2)
- Wales (3)
- Northern Ireland (4)

**Q35** Would you like to share any additional comments and insights on interdisciplinarity in coastal research from an early career researcher's perspective?

________________________________________________________________

Webinar poll

A link to each question was included in the online webinar chat box. Only attendees of the live webinar could respond to the questions.

Questions:

Q1 How would you describe your field/discipline(s)? (open-ended question)

Q2 What is the biggest challenge for you in interdisciplinary research? (multiple choice question)

- Differences (cultural, methodological, vocabularies)
- Systemic issues (fixed/short term contracts, time constraints, academic landscape)
- Proposal requirements (work packages timeline, data dependency)
- Other

Q3 Do you have any other possible solutions? (open-ended question)

Q4 How do you feel about interdisciplinary research? (open-ended question)

Coded excerpts from internal workshops, online survey and webinar polls and discussion.

| **Question** | **Answer** | **Method** | **Theme 1** | **Theme 2** | **Disciplinary differences/Systemic issues/Project Structure** |
| --- | --- | --- | --- | --- | --- |
| **Barrier** | Cultural differences and different backgrounds | Internal workshop | Cultural differences |  | Disciplinary differences |
| **Barrier** | Different vocabularies | Internal workshop | Different epistemologies | Communication | Disciplinary differences |
| **Barrier** | Lack of knowledge of different fields | Internal workshop | Different epistemologies | Professional development | Disciplinary differences |
| **Barrier** | The different level of understanding often causing misunderstanding of the cooperation, but it takes a lot of time to proper understand one concept to the professional level | Online survey - Qualtrics | Different epistemologies | Time | Disciplinary differences |
| **Barrier** | Different methodologies (e.g. use of qualitative or quantitative data) | Internal workshop | Different epistemologies |  | Disciplinary differences |
| **Barrier** | No one understands each other or their backgrounds it takes a lot of work to explain r to social science or participatory research to natural science | Online survey - Qualtrics | Different epistemologies |  | Disciplinary differences |
| **Barrier** | Just continuing along that line of thought, I agree with the perspective shared, so far, but I think I also find myself kind of getting a bit tired in some situations, being that advocate. So my background is interdisciplinary in nature, so I'm fortunate that I can represent marine ecologists and social scientists, but I often find that if I go into a space where I think "oh, this will be great, I haven't done much marine ecology in a while, I'll get to talk about marine ecology" I end up having to be the advocate for thinking about the social aspects of marine ecology and the opposite is sometimes true as well. And so I guess there is kind of a... fatigue if you are an interdisciplinary individual having to always be the voice of the underrepresented disciplines. I would rather do that than not have those voices heard, but I guess communicating to some people, who you know we need people who are specialists and really focus on one discipline, but I guess making sure that those individuals know that they can't ignore the other the other disciplines... And being a bit more mindful that it shouldn't fall on one or two interdisciplinary individuals to take up that task of constantly challenging the status quo in a way, depending on the conversation. | Webinar - discussion | Fatigue - always being an advocate | Power dynamics | Disciplinary differences |
| **Barrier** | Do you sometimes feel imposter syndrome in interdisciplinary spaces? Such as if you were a more scientific person within an economic/social science space | Webinar - discussion | Imposter syndrome |  | Disciplinary differences |
| **Barrier** | The usual "social science isn't a proper science" attitude from natural science collaborators | Online survey - Qualtrics | Lack of appreciation of social scientists |  | Disciplinary differences |
| **Barrier** | Lack of recognition of social science skills and experience within interdisciplinary research teams | Online survey - Qualtrics | Lack of appreciation of social scientists |  | Disciplinary differences |
| **Barrier** | I have experienced - both in networking and projects - a disregard for the years of skills and methodological experience I have as a social scientist involved in interdisciplinary research. In the face of physical science colleagues who think they ' can do social science' because we will JUST do 'some surveys' and claim they are interdisciplinary - this devalues social science rigour and practice and has discouraged me from engaging in some of this important work. | Online survey - Qualtrics | Lack of appreciation of social scientists |  | Disciplinary differences |
| **Barrier** | Project structure does not account for different timelines and data dependency | Internal workshop | Project management |  | Project structure |
| **Barrier** | Time constraints | Internal workshop | Time |  | Project structure |
| **Barrier** | Fixed/short term contracts | Internal workshop | Employment |  | Systemic |
| **Barrier** | Do universities and impactful journals give the same value to Interdisciplinary leaders? The senior successful folk tend to need to be specialists… sadly I think this is part of the core problem! And senior academics are not always willing to try and change the system even though they are in the position of power to do so. | Webinar - chat | Power dynamics | Professional development | Systemic |
| **Barrier** | Requirements for interdisciplinarity but the academic landscape and structures (time/publishing/ career projection) do not go at the same speed | Internal workshop | The gap between the ambition and the reality of interdisciplinary research | Professional development | Systemic |
| **Barrier** | Time constraints | Internal workshop | Time |  | Systemic |
| **Barrier** | I selected 'Other' in the question about challenges because I find that the most challenging aspect for me is the fact that early career researchers like myself don't always get a positive and supportive response from senior collaborators when we push for more innovative interdisciplinary ways of working. I really appreciated hearing that your project believes that new interdisciplinary leaders should be supported! | Webinar - chat | Power dynamics | Professional development | Systemic |
| **Barrier** | The unreasonable competition between PIs | Online survey - Qualtrics | Competing interests |  | Systemic issue |
| **Barrier** | We need to promote an open and friendly atmosphere in the ocean and climate academic community to jointly address the challenges of global change, rather than engaging in unhealthy competition between PIs and labs. | Online survey - Qualtrics | Competing interests |  | Systemic issue |
| **Barrier** | Institutional demand management in UKRI funding limits opportunities for grant dev/submission required for continued employment possibilities, hiring delays between contracts make postdoc roles financially elitist even when funding is obtained | Online survey - Qualtrics | Employment | Funding opportunities | Systemic issue |
| **Barrier** | Short term contracts and maternity leave periods mean there is still a glass ceiling for women in practical terms | Online survey - Qualtrics | Employment | Gender | Systemic issue |
| **Barrier** | The gap between ECR and the next step up in career e.g. gaining a fellowship | Online survey - Qualtrics | Employment | Lack of professional development opportunities | Systemic issue |
| **Barrier** | Lack of support for young researchers to recruit PhD students and postdocs | Online survey - Qualtrics | Lack of professional development opportunities | Power dynamics | Systemic issue |
| **Barrier** | Lack of networking opportunities with like-minded people to collaborate within ourselves. | Online survey - Qualtrics | Lack of professional development opportunities |  | Systemic issue |
| **Barrier** | suitable training and courses | Online survey - Qualtrics | Lack of professional development opportunities |  | Systemic issue |
| **Barrier** | Developing relevant contacts if field differs from supervisor can be difficult | Online survey - Qualtrics | Lack of professional development opportunities |  | Systemic issue |
| **Barrier** | Academia is a shit show. Most people don't understand what interdisciplinary or transdisciplinary work is like. | Online survey - Qualtrics | Lack of understanding of interdisciplinary research |  | Systemic issue |
| **Barrier** | Interdisciplinary is like hybrid, rare and unique unfortunately not most of the market demand understands this concept. I'd say the society needs the change of mindset. | Online survey - Qualtrics | Lack of understanding of interdisciplinary research |  | Systemic issue |
| **Barrier** | Mental health | Online survey - Qualtrics | Mental health |  | Systemic issue |
| **Barrier** | interdisciplinary researchers most likely to involve different scientists from different backgrounds with different characters. We should respect each other and seek common ground while reserving differences in order to achieve long-term cooperation. Young scientists' voices are particularly vulnerable to lack of respect from authority, and open communication is often seen as arrogant. | Online survey - Qualtrics | Power dynamics |  | Systemic issue |
| **Barrier** | Time | Online survey - Qualtrics | Time |  | Systemic issue |
| **Barrier** | Lack of great harness the interdisciplinary research could present, such as innovation solutions | Online survey - Qualtrics | The gap between the ambition and the reality of interdisciplinary research |  | Systemic issue/Project structure |
| **Barrier and solution** | I am an archaeologist who works on interdisciplinary projects with climate scientists and coastal engineers, including modellers. For me, the most important thing is respect across disciplines. I am not sure that everyone feels that everyone else's discipline is as rigorous as the hard sciences. I find that scientists feel they understand and can 'do' the archaeology and heritage elements without the years of training that comes with it. But I have found it just takes disciplinary respect and good communication | Webinar - chat | Communication | Respect | Disciplinary differences |
| **Barrier and solution** | That people feel like disciplinary outcasts, that kind of rang with me. I like that term and I hate that term, because maybe I come from a background of social science and am now surrounded by the marine environment… adapting learning new things every day, you kind of feel like an outcast even though you are being welcomed. So it is just interesting to hear that being said by someone else. Maybe this is also a good solution, just to exchange these experiences and know, okay others feel the same way, but there are solutions. | Webinar - discussion | Shared experience of being an outsider | Power dynamics | Disciplinary differences |
| **Solution** | Sometimes the outsider is the one with knowledge that the others don't have and it can be difficult to put that forward. I've been in that situation as a biologist talking to sedimentoligists… and we all make assumptions about our science, but actually it's the outsider that sometimes comes in and goes "well actually that's wrong because you haven't thought of this". So I think Chrissy is right, you know you can feel like the outcast, but often the outcast has quite a lot of value. | Webinar - discussion | Value of different viewpoints |  | Disciplinary differences |
| **Barrier and solution** | Finally finding a permanent position has been the greatest boost in my career, it allowed me to stop losing time for applications for jobs and focus on my research, plus it gave me the enthusiasm to invest in my research community without the stress of feeling disposable. Short term contracts are a huge fault of the research system that fail a lot of talented young researchers eventually pushing them out of research. I'm lucky to work for an employer that values the input of young researchers but in previous experiences hierarchy has discouraged me and hindered my capacity to explore new research avenues and develop my interests. | Online survey - Qualtrics | Employment | Power dynamics | Systemic |
| **Benefit** | Open minded when assessing projects, and capable of uniting many differing skillsets effectively. | Online survey - Qualtrics | Holistic approach |  | Disciplinary differences |
| **Benefit** | It conveys the whole picture, the marine environment is not on its own | Online survey - Qualtrics | Holistic approach |  | Disciplinary differences |
| **Benefit** | One can see more angles of research and tackle the problem using diverse methodologies. Also, using environmental science only and forgetting about the sociocultural aspects as well as stakeholder involvement is a miss because coastal/marine research has major implications for humans. | Online survey - Qualtrics | Holistic approach |  | Disciplinary differences |
| **Benefit** | gives the opportunity for whole system approaches in a liminal space | Online survey - Qualtrics | Holistic approach |  | Disciplinary differences |
| **Benefit** | The coastal environment is complex and interactions need to be considered. | Online survey - Qualtrics | Holistic approach |  | Disciplinary differences |
| **Benefit** | Better understanding of the system, taking into account the complexity of the problem, accounting for local communities' perspectives | Online survey - Qualtrics | Holistic approach |  | Disciplinary differences |
| **Benefit** | I view the coastal areas as inherently interdisciplinary because of its proximity to human activity. Processes are naturally linked (ecology-fisheries-society-economy etc.) | Online survey - Qualtrics | Holistic approach |  | Disciplinary differences |
| **Benefit** | I think interdisciplinary research is really vital in the sense that we're looking at a view of the impact on human communities and as biologists or as science achievers, we've got to actually bring in the society aspect of things and the implications and the impact which it has on communities. So I think from that point of view we've got to learn from the social scientists a lot, because they can bring in a totally different perspective, but also engaging with the natural, like climate change or natural disasters, and we are getting more and more into events of high intensity of flooding - I do a lot of work in Pakistan and the rural communities there are really impacted and they have to be uplifted in some way or given the resources to defend themselves in these environments. So I think we have to, it's a team effort, so we have experts say hydrologists, engineers, biologists and also social scientists interacting together and creating a better result from the studies we do. So interdisciplinary research is very much needed. And we of course learn from each other and I think the vocabulary is a very good example where we have differences, because I talked about indigenous communities and somebody else from humanities background talked of it differently, so again coming to a compromise or a standard definition, I think this is also meeting the criteria or the aims of our studies | Webinar - discussion | Important for tackling complex challenges/**Community benefits** | Communication/different epistemologies | Disciplinary differences |
| **Benefit** | expansion of data resources, capacity building, increased knowledge and more outputs such as papers | Online survey - Qualtrics | *Knowledge exchange* | Professional development/Value for money | Disciplinary differences/systemic |
| **Benefit** | There are several benefits but two main ones in my opinion are knowledge exchange and greater impact on the work with an interdisciplinary approach | Online survey - Qualtrics | Knowledge exchange | Value for money | Disciplinary differences/systemic |
| **Benefit** | Increases efficiency of funding for tackling issues/questions working together instead of separately | Online survey - Qualtrics | Value for money |  | Project structure/Systemic |
| **Benefit** | Create additional avenues for alternative funding. | Online survey - Qualtrics | Funding opportunities | New opportunities | Systemic |
| **Benefit** | It's much more holistic. All well and good creating solutions for nature but unless people are included equally nothing will happen (or things will happen with resentment). Also, the opportunities for those of us that have moved over to interdisciplinary research are huge! | Online survey - Qualtrics | Holistic approach | New opportunities | Systemic |
| **Benefit** | Multi-dimensional stability always strengthen the mitigation measures and ensure the sustainability in the ecosystem. | Online survey - Qualtrics | Holistic approach | Sustainability | Systemic |
| **Benefit** | To understand | Online survey - Qualtrics | Holistic approach |  | Systemic |
| **Benefit** | more impactful outputs | Online survey - Qualtrics | *Impact* |  | Systemic |
| **Benefit** | Sustainable development | Online survey - Qualtrics | Sustainability |  | Systemic |
| **Benefit - career** | Science doesn’t happen in silos | Online survey - Qualtrics | Holistic approach |  | Disciplinary differences |
| **Benefit - career** | It strengthens research but drawing on multiple perspectives, methodologies and philosophies | Online survey - Qualtrics | Holistic approach |  | Disciplinary differences |
| **Benefit - career** | new perspectives bring new approaches and learning. | Online survey - Qualtrics | Professional development |  | Disciplinary differences |
| **Benefit - career** | It keeps me engaged in learning and expanding my research horizon | Online survey - Qualtrics | Professional development |  | Disciplinary differences |
| **Benefit - career** | Gives more transferable skills. Improves ability to think flexibly and out-of-the-box. | Online survey - Qualtrics | Professional development |  | Disciplinary differences |
| **Benefit - career** | More employable | Online survey - Qualtrics | Employment |  | Systemic |
| **Benefit - career** | More job opportunities (it’s easier to change discipline if needed) | Online survey - Qualtrics | Employment |  | Systemic |
| **Benefit - career** | Has allowed pivoting in areas where funding is a 'hot topic' | Online survey - Qualtrics | Funding opportunities | New opportunities | Systemic |
| **Benefit - career** | Its a buzzword | Online survey - Qualtrics | Funding opportunities |  | Systemic |
| **Benefit - career** | Clearer impact of science | Online survey - Qualtrics | Impact |  | Systemic |
| **Benefit - career** | It may [be] less 'competitive' | Online survey - Qualtrics | Professional development | Employment | Systemic |
| **Benefit - career** | I've been a interdisciplinary researcher and I believe it helps to upskill oneself to progress self actualization while giving optimum benefits to the society and environment. | Online survey - Qualtrics | Professional development | Impact | Systemic |
| **Benefit - career** | Learning, broadening horizons | Online survey - Qualtrics | Professional development |  | Systemic |
| **Benefit - career** | Opens doors to opportunities and is a lot more meaningful (less siloed thinking) | Online survey - Qualtrics | Professional development | Holistic approach | Systemic/Disciplinary differences |
| **Benefit - career** | Scientific research is a career that requires lifelong learning. Our natural sciences come from nature, and all things in nature are interconnected. Interdisciplinary learning can easily find breakthroughs and innovations through connections. | Online survey - Qualtrics | Professional development | Holistic approach | Systemic/Disciplinary differences |
| **Solution** | Regular meetings between PDRAs and others to talk, reflect and understand each other | Internal workshop | Communication | Team building | Disciplinary differences |
| **Solution** | Approach it with the attitude of ""I don't know everything!" | Webinar - polls | Humility | Respect | Disciplinary differences |
| **Solution** | Programmes such as SMMR subject to learning from current projects and reflect and change for their future rounds | Internal workshop | Impact | Funding opportunities | Disciplinary differences |
| **Solution** | Taking time to share one's work with others (and taking time to engage) | Internal workshop | Knowledge exchange | Communication | Disciplinary differences |
| **Solution** | Attending training and networking opportunities such as SMMR organised training on interdisciplinary research and Ocean Partnerships | Internal workshop | Professional development |  | Disciplinary differences |
| **Solution** | Map different objectives from each of the disciplines and prioritize based on a timeline following the overall goals | Webinar - polls | Project management |  | Disciplinary differences/project structure |
| **Solution** | Integrating interdisciplinary view at an early stage (e.g schools) | Webinar - polls | Education |  | Disciplinary differences/systemic |
| **Solution** | I believe that there is not so much a need for interdisciplinarity for its own sake, as for those who are (in some sense) specialists to make an effort to see their own work in wider contexts. This is a notable feature of the best contemporary research grant proposals. | Webinar - chat | Open-minded | Funding opportunities | Disciplinary differences/systemic |
| **Solution** | Creating a framework with unified terminology and methods before beginning project. | Webinar - polls | Communication | Project management | project structure |
| **Solution** | Meet in person | Webinar - polls | Communication | Team building | project structure |
| **Solution** | Frequent communication. | Webinar - polls | Communication |  | project structure |
| **Solution** | Have contingency plans and flexibility in deliverables | Internal workshop | Project management |  | Project structure |
| **Solution** | Have an experienced “Interdisciplinary Champion” | Internal workshop | Project management |  | Project structure |
| **Solution** | Communication is key. Be honest with timescales and capabilities from the start of the project. If someone doesn't understand the technicalities of what you are working on they may also not know the timescales and the capabilities. | Webinar - polls | Communication | Project management | project structure/disciplinary differences |
| **Solution** | Fund project development phases not just post-objective setting. | Webinar - polls | Funding opportunities |  | Systemic |
| **Solution** | I think it’s an exciting field that will grow -- there is a need for capacity building, training ECRs in interdisciplinary approaches and inviting their own ideas to the mix | Online survey - Qualtrics | Professional development |  | Systemic |
| **Solution** | Work packages/tasks should have joint leadership, with people from different disciplines to ensure better integration of tasks throughout the project | Internal workshop | Project management |  | Systemic |
| **Solution** | I think multidisciplinary scientists are well placed in coastal research due to its complex nature, and interface between land and sea. Having an open minded approach utilising multiple methods allows for greater understanding of these systems, further willing ness to collaborate, and ability to see the larger picture (this is not to say fine scale research is also not valuable, both work effectively alongside each other!) | Online survey - Qualtrics | Holistic approach | Open-minded | Systemic/Disciplinary differences |
| **Solution** | Be good to use fair work contracts to engage with freelancers so they can be paid fairly for all time and expertise (and to be specific about in-kind support) | Webinar - polls | Employment |  | systemic/project structure |
